# Inequalities in health-related quality of life: repeated cross-sectional study of trends in general practice survey data

**DOI:** 10.3399/BJGP.2020.0616

**Published:** 2021-02-23

**Authors:** Vishalie Shah, Jonathan Stokes, Matt Sutton

**Affiliations:** Centre for Health Economics, University of York, York; Health Organisation, Policy and Economics (HOPE), Centre for Primary Care and Health Services Research, University of Manchester, Manchester.; HOPE, Centre for Primary Care and Health Services Research, University of Manchester, Manchester.; HOPE, Centre for Primary Care and Health Services Research, University of Manchester, Manchester.

**Keywords:** mental health, population health, public health, quality of life, socioeconomic factors

## Abstract

**Background:**

After decades of steady progress, life expectancy at birth has stalled in England. Inequalities are also rising, and life expectancy has fallen for females living in the most deprived areas. However, less attention has been given to trends in other measures of population health, particularly health-related quality of life (HRQoL).

**Aim:**

To examine trends and inequalities in HRQoL in England between 2012 and 2017.

**Design and setting:**

The authors used nationally representative survey data on 3.9 million adults to examine HRQoL (measured by EQ-5D-5L overall score, plus each of the five health domains — mobility, selfcare, usual activity, pain/discomfort, and anxiety/depression).

**Method:**

The study explored trends across time, and inequalities by sex, age, and deprivation.

**Results:**

Although HRQoL seemed steady overall between 2012 and 2017, there is evidence of increasing inequality across population subgroups. There was a rise in sex disparity over time, the female–male gap in EQ-5D-5L increased from −0.009 in 2012 to −0.016 in 2017. Trends for the youngest females and those living in the most deprived areas were of the greatest concern. Females in the most deprived regions suffered a 1.3% decrease in HRQoL between 2012 and 2017, compared with a 0.5% decrease for males. The key contribution to the decline in HRQoL, particularly in females, was a 1.5% increase in reported levels of anxiety/depression between 2012 and 2017.

**Conclusion:**

Developing interventions to address these worrying trends should be a policy priority. A particular focus should be on mental health in younger populations, especially for females and in deprived areas.

## INTRODUCTION

After decades of steady progress, life expectancy at birth in England has stalled.^1^ This trend is also apparent in other high-income countries, such as the US.^2^ Health inequalities have also increased, with falling life expectancy among females living in deprived areas.^3^ Though there is close monitoring of life expectancy, little attention is paid to other measures of population health.

There are reasons for this predominant focus on life expectancy at birth. It is a routinely available indicator of population health and can be used to make comparisons over time and across health systems.^4^ However, it fails to reflect variations in the quality of life while people are alive.^5^ It is sometimes adapted to ‘healthy life expectancy’, which adds an adjustment based on the proportion of people reporting ‘very good’ or ‘good’ general health.^6^ This is crude, prone to reporting bias, and does not reveal which specific aspect(s) of health are contributing to compromised quality of life.^7^^,^^8^

Health-related quality of life (HRQoL) is an alternative, multidimensional measure of health that incorporates physical, mental, and social domains of health into a single figure. Arguably, it therefore better reflects the World Health Organization’s (WHO) definition of health, *‘a state of complete physical, mental, and social wellbeing and not merely the absence of disease or infirmity’*.^9^ HRQoL and life expectancy can move in similar or opposite directions. If HRQoL is falling while life expectancy is constant, this would indicate an overall decrease in population health. However, if HRQoL is still rising, overall population health might still be improving.

The authors used nationally representative survey data from England to examine how HRQoL changed between 2012 and 2017. They used the EQ-5D-5L, the measure of HRQoL that is preferred by the National Institute for Health and Care Excellence (NICE) in the UK, and similar institutions in other regions, to inform policymaking and purchasing decisions.^10^^–^^13^ The authors explored trends across each of the five dimensions of health and inequalities by population subgroups — sex, age, and deprivation — comparable with the related life expectancy literature.^14^

**Table:** How this fits in

Although life expectancy at birth has stalled in some high-income countries and inequalities have widened, there has been less focus on other population health indicators, such as health-related quality of life (HRQoL). The authors examine trends and inequalities in HRQoL in England using data on 3.9 million adults from large national surveys from 2012 to when the series ended in 2017. There has been no change in average HRQoL, but there have been increases in inequalities for females, particularly the youngest and those living in the most deprived areas. These deteriorations are driven by increases in anxiety and depression, and should be a future policy priority.

## METHOD

### Data

The authors used the General Practice Patient Survey (GPPS) (https://www.gp-patient.co.uk), an independent, national patient survey conducted by Ipsos MORI. The GPPS is sent by post to 2 million randomly selected patients from all GP practices in England, providing they are aged ≥18 years, have an NHS number, and have been registered with a GP practice for 6 months. All responders are anonymous and cannot be followed over time. The GPPS was conducted bi-annually between 2012 and 2015, and annually from 2016 onwards. Bi-annual data were collected from January–March and July–September of each year; annual data were collected from January–March of each year. Survey weights that account for the sampling design and the impact of non-response bias ensure results are representative of the population of adult patients registered with a GP.

The authors obtained GPPS data from nine survey waves (collected between July–September 2012 and January–March 2017) at the individual-patient level through NHS England, and extracted information on patients’ EQ-5D-5L responses. The most recent five-level (5L) version, introduced in 2011, allows responders to choose between five levels across each dimension: no problems, slight problems, some problems, severe problems, or extreme problems.^15^ The level selected for each dimension is combined with a value set to produce a single index value.^16^ EQ-5D-5L scores are measured on a scale between −0.59 to 1, where 1 indicates perfect health, 0 indicates death, and negative values indicate a state worse than death.

The authors additionally extracted data on sex, age, area deprivation, and region. Area deprivation was measured by the Index of Multiple Deprivation.^17^

For mapping purposes, the authors obtained administrative data from the Office for National Statistics, including look-up files for geographical boundaries,^18^ and the latest digital vector boundaries for clinical commissioning groups (as at April 2018) from the Open Geography Portal.^19^

From an initial set of 4 378 022 responders, 1230 (0.003%) observations that did not have information on responders’ local authority district were deleted, as were 438 476 (10%) observations with incomplete data on EQ-5D-5L, age, sex, or deprivation; 3 938 316 observations remained.

### Analyses

The authors plotted the average of the EQ-5D-5L score for the full sample population between 2012 and 2017. They then examined health inequalities. First, they examined sex differences in trends of EQ-5D-5L for males and females separately, and additionally for sex-specific age categories. Second, they examined socioeconomic inequalities by dividing responders into quintiles of area deprivation, and tracking the course of EQ-5D-5L for males and females. Third, to examine changes at a geographical level, the authors used digital vector boundaries and mapping software to create choropleth maps that visualised the spatial distribution of the change in EQ-5D-5L across clinical commissioning groups between 2012 and 2017. Finally, they examined the trajectory of each of the five domains in the EQ-5D-5L separately (scored 1 to 5 representing the five levels) to explore whether particular domains were driving the overall trend. In each line graph, the authors displayed error bars that represent 95% confidence intervals (CIs). Stata/MP (version 16) and R (version 3.6.3) were used.

## RESULTS

### Sample characteristics

The weighted sample characteristics were largely similar over time (Table 1). Just over half of the responders were female, and about 20% were >65 years old. The mean EQ-5D-5L score was around 0.8, with an average level better than 4 (‘slight problems’) in each domain.

**Table 1.** Sample characteristics of GPPS responders across England for each survey waves

	**Jul–Sep 2012**		**Jan–Mar 2013**		**Jul–Sep 2013**		**Jan–Mar 2014**		**Jul–Sep 2014**		**Jan–Mar 2015**		**Jul–Sep 2015 Jan–Mar 2016**				**Jan–Mar 2017**	
**Demographic characteristics^a^**																		
% female	0.51	(0.50)	0.51	(0.50)	0.51	(0.50)	0.50	(0.50)	0.51	(0.50)	0.51	(0.50)	0.51	(0.50)	0.51	(0.50)	0.51	(0.50)
% aged 18–24	0.10	(0.30)	0.10	(0.30)	0.10	(0.30)	0.10	(0.30)	0.10	(0.30)	0.10	(0.29)	0.10	(0.30)	0.10	(0.30)	0.09	(0.29)
% aged 25–34	0.18	(0.38)	0.18	(0.38)	0.18	(0.38)	0.18	(0.38)	0.17	(0.38)	0.17	(0.38)	0.18	(0.38)	0.18	(0.38)	0.17	(0.38)
% aged 35–44	0.18	(0.39)	0.18	(0.38)	0.18	(0.38)	0.18	(0.38)	0.17	(0.38)	0.18	(0.38)	0.17	(0.38)	0.17	(0.38)	0.17	(0.38)
% aged 45–54	0.19	(0.39)	0.19	(0.39)	0.19	(0.39)	0.19	(0.39)	0.19	(0.39)	0.19	(0.39)	0.19	(0.39)	0.19	(0.39)	0.19	(0.39)
% aged 55–64	0.15	(0.36)	0.15	(0.35)	0.15	(0.36)	0.15	(0.36)	0.15	(0.36)	0.15	(0.36)	0.15	(0.36)	0.15	(0.36)	0.15	(0.36)
% aged 65–74	0.11	(0.32)	0.12	(0.32)	0.12	(0.32)	0.12	(0.32)	0.12	(0.33)	0.12	(0.33)	0.12	(0.33)	0.12	(0.33)	0.13	(0.33)
% aged 75–84	0.07	(0.25)	0.07	(0.25)	0.07	(0.25)	0.07	(0.25)	0.07	(0.25)	0.07	(0.25)	0.07	(0.25)	0.07	(0.25)	0.07	(0.25)
% aged ≥85	0.02	(0.16)	0.03	(0.16)	0.03	(0.16)	0.03	(0.16)	0.03	(0.16)	0.03	(0.16)	0.03	(0.16)	0.03	(0.16)	0.03	(0.16)
IMD 2015	22.00	(15.65)	21.58	(15.45)	21.62	(15.53)	21.64	(15.52)	21.64	(15.53)	21.64	(15.49)	21.62	(15.50)	21.69	(15.48)	21.70	(15.51)
**EQ-5D-5L^a^**																		
Utility score^b^	0.821	(0.23)	0.825	(0.22)	0.821	(0.23)	0.822	(0.22)	0.822	(0.22)	0.823	(0.23)	0.823	(0.22)	0.820	(0.23)	0.818	(0.23)
Mobility	4.593	(0.85)	4.612	(0.83)	4.594	(0.85)	4.607	(0.84)	4.597	(0.84)	4.610	(0.83)	4.604	(0.84)	4.612	(0.83)	4.612	(0.83)
Self-care	4.842	(0.57)	4.844	(0.57)	4.843	(0.57)	4.843	(0.57)	4.847	(0.56)	4.843	(0.57)	4.846	(0.57)	4.844	(0.57)	4.840	(0.58)
Usual activities	4.566	(0.87)	4.583	(0.86)	4.568	(0.87)	4.577	(0.86)	4.573	(0.86)	4.581	(0.86)	4.578	(0.86)	4.580	(0.86)	4.580	(0.86)
Pain/discomfort	4.272	(0.93)	4.295	(0.92)	4.274	(0.93)	4.281	(0.92)	4.277	(0.93)	4.285	(0.92)	4.283	(0.93)	4.279	(0.92)	4.274	(0.93)
Anxiety/depression	4.533	(0.82)	4.535	(0.82)	4.532	(0.83)	4.524	(0.82)	4.536	(0.82)	4.520	(0.83)	4.530	(0.83)	4.503	(0.85)	4.482	(0.86)
*N*	425 734		442 215		400 629		409 923		381 628		390 748		378 241		376 764		732 434	

a Data presented as weighted mean (SD).

b Utility score of 1 indicates perfect health. GPPS = General Practice Patient Survey. IMD = Index of Multiple Deprivation. SD = standard deviation.

### Visual graphs

**Figure 1.**
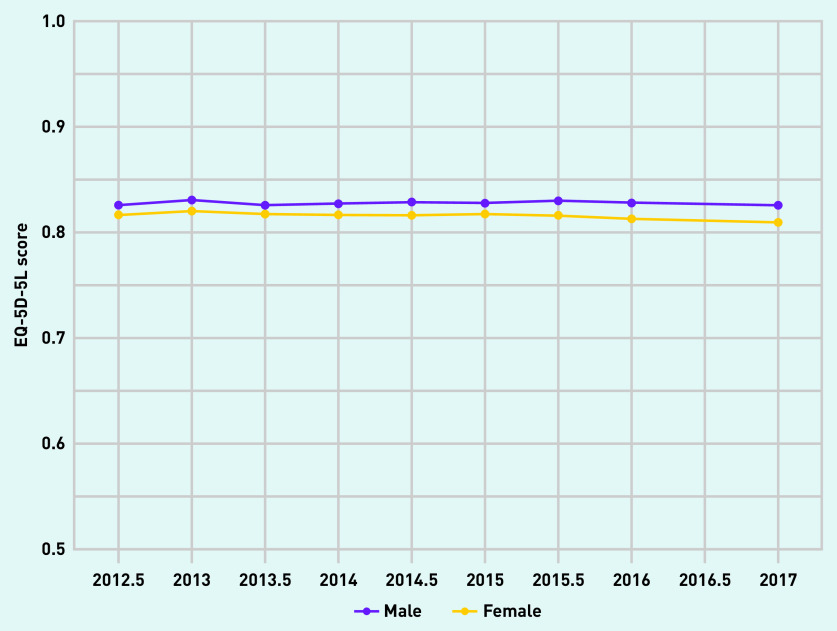
***EQ-5D-5L score (with 95% confidence intervals) for males and females, England, 2012–2017.***

Figure 1 shows that the average EQ-5D-5L score was fairly static between 2012 and 2017, with a slight downward trend. EQ-5D-5L for males was greater than that for females at every timepoint. The error bars do not overlap, confirming the difference is statistically significant, but the absolute difference is small. From 2015 onwards, however, the trends begin to deviate slightly — HRQoL begins to fall at a steeper rate for females, leading to increasing sex inequalities. Overall, the mean EQ-5D-5L score for males was 0.826 (95% CI = 0.824 to 0.827) in 2012 and 0.826 (95% CI = 0.825 to 0.827) in 2017; for females, the mean EQ-5D-5L score was 0.817 (95% CI = 0.816 to 0.818) in 2012 and 0.810 (95% CI = 0.809 to 0.811) in 2017. Thus the female–male gap in EQ-5D-5L increased from −0.009 in 2012 to −0.016 in 2017.

**Figure 2.**
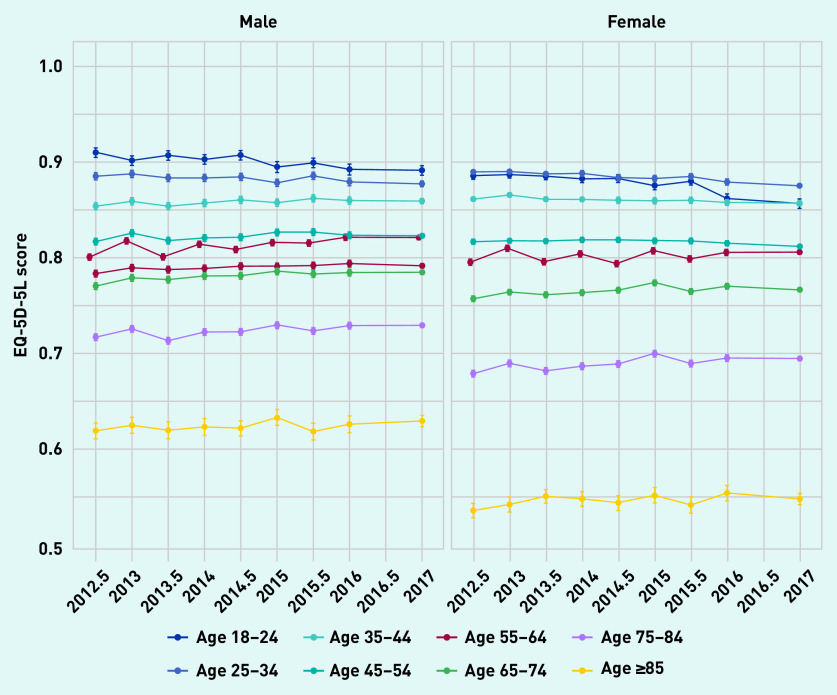
***EQ-5D-5L score (with 95% confidence intervals) for males and females by age categories, England, 2012–2017.***

Figure 2 explores how much of the sex variation in EQ-5D-5L was attributed to responders’ age. As expected, there was a laddered effect by age band, with younger individuals reporting higher average HRQoL. The average EQ-5D-5L scores were similar between males and females in the younger age groups. However, from age ≥65 years there were progressively larger gaps, with higher scores for males than females. It is likely that this at least partially reflects the higher average length of life for females.

The trends for almost all age bands were steady for both age and sex groups over time. However, the exception was the trend for young females, particularly those aged 18–24 years, whose average EQ-5D-5L score dropped from 0.887 (95% CI = 0.884 to 0.891), which was equivalent to females aged 25–34 years in 2012, to 0.858 (95% CI = 0.854 to 0.862), which was equivalent to females aged 35–44 years by 2017.

**Figure 3a.**
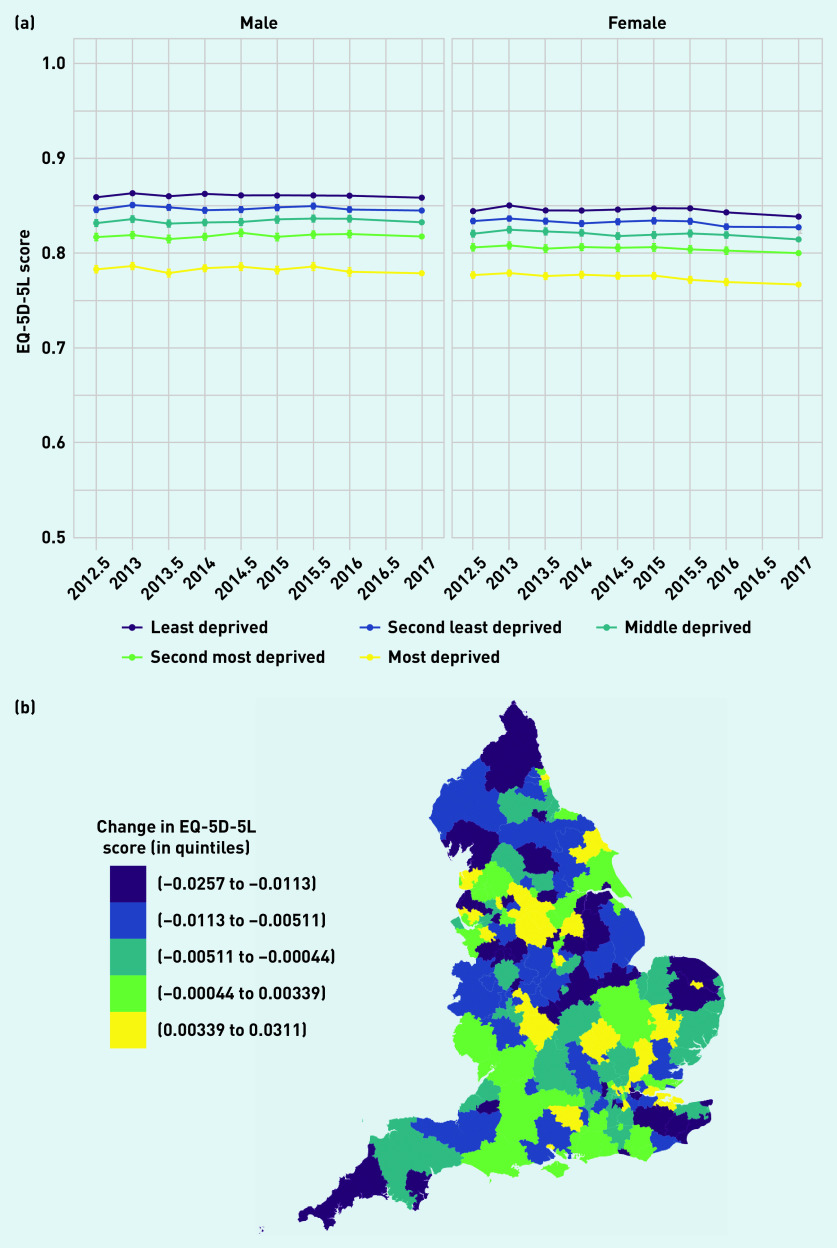
***EQ-5D-5L score (with 95% confidence intervals) for males and females by quintiles of deprivation, England, 2012–2017. 3b. Change in EQ-5D- 5L score across clinical commissioning groups by quintiles, England, 2012–2017.***

Figure 3 shows inequalities in HRQoL by area deprivation. For every quintile of deprivation, females reported a lower EQ-5D-5L than males across all time periods. The average scores were statistically equivalent for all deprivation quintiles for males in 2012 and 2017 but decreased for all deprivation quintiles of females. The sharpest decline occurred in the most deprived females, with the average EQ-5D-5L score falling from 0.777 (95% CI = 0.774 to 0.780) in 2012 to 0.767 (95% CI = 0.765 to 0.769) in 2017, a decline of 1.3%. Declines in EQ-5D-5L have also occurred mainly in deprived regions,^6^ such as the North and the Midlands.

**Figure 4.**
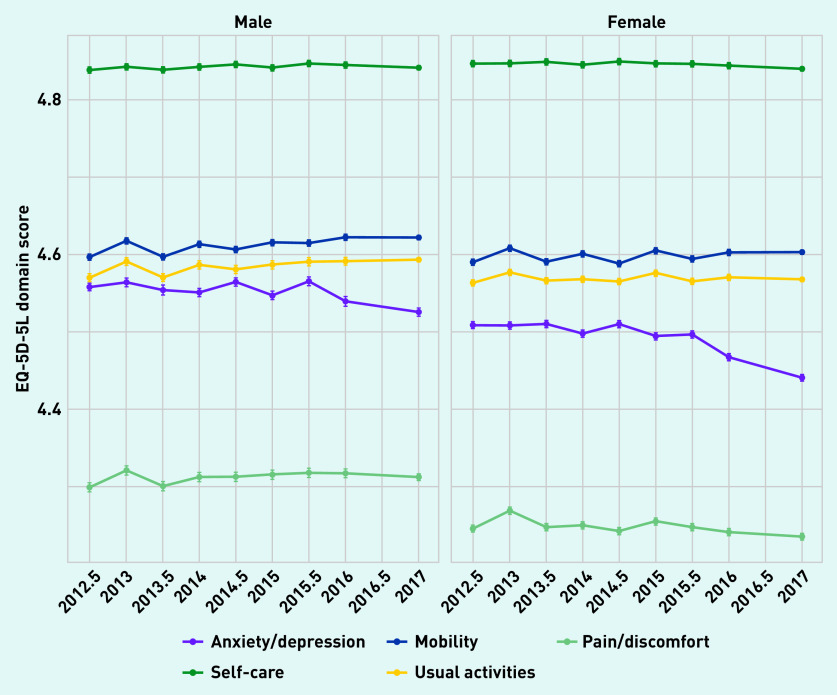
***EQ-5D-5L domain score (with 95% confidence intervals) for males and females, England, 2012–2017.***

Figure 4 shows the extent to which the trajectory of EQ-5D-5L can be explained by each of its five domains. Consistently, self-care was the highest reported domain, and pain/discomfort was the lowest reported domain across the population. Responders’ scores for self-care, usual activities, and mobility were largely similar across sex and time. However, anxiety/depression and pain/discomfort scores were worse for females than males for all time periods. Furthermore, anxiety/depression scores gradually worsened over time, particularly for females, with a decline in the average domain score from 4.509 (95% CI = 4.505 to 4.513) in 2012 to 4.441 (95% CI = 4.369 to 4.444) in 2017, a difference of 1.5%. This decline was more pronounced from 2015 onwards.

## DISCUSSION

### Summary

The results show that, although EQ-5D-5L seemed to be steady overall between 2012 and 2017, there is evidence of increasing inequality in HRQoL across population subgroups. The authors identified disparities in EQ-5D-5L across sex. Overall, males reported higher scores than females at each timepoint in the study, and this disparity increased from 2015 onwards. The younger female population (aged 18–24 years) accounted for this increasing inequality, as they reported the largest decline in HRQoL over time. The most deprived quintile of the population had the lowest HRQoL at every timepoint; in particular, deprived females reported the lowest EQ-5D-5L score across the population that worsened over time. The most deprived regions suffered decreases in HRQoL over time, while wealthier regions improved. The key driver of the decline in EQ-5D-5L over time was increasing levels of anxiety and depression.

### Strengths and limitations

The authors analysed the well-known and widely used EQ-5D-5L instrument, whose reliability as a tool for measuring and valuing health status has been supported by decades of evidence-based research.^20^ They used large-scale, nationally representative data that monitored the trajectory of EQ-5D-5L across 3.9 million responders.

The study was unable to include recent GPPS surveys (2018 and 2019) in the analyses as questions on EQ-5D-5L were removed, and previous years of GPPS used alternative sampling methods (before 2012) or measures of HRQoL (early 2012). The study is therefore restricted to survey waves between mid-2012 and 2017.

The 2016 and 2017 GPPS were conducted in the winter period between January and March. Earlier surveys from 2012–2015 included an additional summer data collection between July and September. The current study’s results, however, display a similar trend during 2016 and 2017 as per the previous years.

### Comparison with existing literature

The results support the growing evidence base that health inequalities across certain population subgroups are widening. In particular, the authors’ findings echo those reported in the recent follow-up to the Marmot Review, that social determinants of health such as sex, region, and socioeconomic circumstances play a large role in determining health.^3^

The gender gap in life expectancy has been a common feature of mortality trends for many years, both in England and across the world more generally, with females living longer lives than males.^21^^,^^22^ It is also widely reported that females spend more of these life-years in poor health, and so the gender gap in healthy life expectancy is relatively smaller.^23^^,^^24^ Previous research suggests that this sex differential in health is not only attributed in part to increased female longevity, but also to structural differences in fundamental characteristics between males and females, and their respective roles in society.^25^^,^^26^

Some studies have additionally explored gender inequality in HRQoL, finding that, on average, females tend to report lower scores than males. After adjusting for functional disability, differences in self-reported health persist as a result of differences in sociodemographic and socioeconomic characteristics, such as age, race, education, and income.^27^^–^^29^ The authors’ results show that, although females overall are more likely than males to experience day-to-day health-related limitations that adversely impact their quality of life, there are particular domains — such as selfcare, mobility, and usual activity — for which they report equivalent scores. Conversely, their scores for pain/discomfort and anxiety/depression are lower than for males. These findings are in line with the evidence that males are more likely to experience life-threatening health shocks that adversely impact their ability to perform daily tasks, whereas females are more likely to experience chronic but non-life-threatening disorders that test their pain threshold and mental health.^30^

The social gradient in health has been a major area of research for many decades.^31^^,^^32^ It is well understood that health inequalities, as a result of socioeconomic factors, can heavily influence life expectancy and healthy life expectancy.^33^ Recent evidence suggests that these inequalities are widening further, and disproportionately affect females living in deprived communities.^3^ The authors’ findings show that from 2015 onwards females living in the most deprived areas experienced a worsening HRQoL, while the situation over the same time period was steady for males. The most deprived areas in this study sample were regions located in the North and the Midlands, which experienced the most negative changes in EQ-5D-5L scores over time, thus reinforcing the already well-established North–South divide in inequalities in health.^34^^–^^36^

An alarming finding is that the youngest males and, particularly, females were the main subpopulations to experience a fall in HRQoL between 2012 and 2017. The driving factor was an increase in anxiety and depression. Mental health disorders are a large contributor to the overall health of young people, and a key determinant of disability and mortality, both in youth and later life.^37^^–^^39^ In the UK, the reported prevalence of affective disorders in young people is rising considerably, with young females being the most impacted.^40^^–^^42^ A growing body of research has evaluated potential determinants of mental health disorders, particularly among young adults, finding that, in addition to well-recognised social and economic risk factors, the current generation of young adults is faced with a novel range of problems relating to social media, educational pressures, financial uncertainty, and changing cultural norms.^43^ Some of these risk factors — for example, the rising psychological distress among students and graduates — may disproportionately affect young females compared with young males.^44^^,^^45^ Further, young people in general are less likely to seek medical help during an emotional crisis, which may explain the worsening trend in anxiety and depression over time if mental health concerns are not addressed.^46^ Ultimately, poor mental health could also be a mechanism for decreasing life expectancy if it leads to suicide. There has been a significant increase in the suicide rate in recent years for both young males and young females; despite low overall number of deaths, the rate has increased by 83% since 2012 for females aged 10–24 years.^47^

### Implications for research and practice

Slowing improvements in life expectancy and widening health inequalities have prompted concerns about the progress of society as a whole.^3^ This study suggests that, although policymakers should continue to understand the main drivers behind the trend in longevity and healthy life expectancy, some additional thought should be given to address similar trends in HRQoL.

Several studies have explored the potential reasons behind the 2015 fall in life expectancy and the slowing of improvements in mortality post-2011. Many have linked these changes to the government austerity policies from 2010 onwards that resulted in reductions of health, social care, and other public budgets likely to affect the social determinants of health.^1^^,^^48^^,^^49^ Others attribute these changes to the increased prevalence of influenza, or even the possibility that life expectancy has reached its physical limits.^1^ The drivers behind these trends are still unclear; however, it is likely that some of the factors that affect mortality also affect quality of life or, as previously highlighted, quality of life can be directly linked to mortality.

An additional finding from this study that warrants attention is that of declining HRQoL in young adults, which seems to be linked to rising mental health issues. There are ongoing concerns over the increased prevalence of mental health problems in England, the widening gap in mental health inequalities, and the potential link to welfare policies and austerity measures as the main contributing factors.^50^^,^^51^ Developing interventions to address these worrying trends should be a policy priority.

Future research could further investigate health inequalities by exploring trends in EQ-5D-5L by ethnicity, for example, given the availability of this data in the GPPS.
